# The Impact of Multidimensional Poverty on Rural Households’ Health: From a Perspective of Social Capital and Family Care

**DOI:** 10.3390/ijerph192114590

**Published:** 2022-11-07

**Authors:** Hui Xiao, Xian Liang, Chen Chen, Fangting Xie

**Affiliations:** 1 School of Economics and Management, Zhejiang A & F University, Hangzhou 311300, China; 2 School of Economics and Management, Beijing Forestry University, Beijing 100083, China; 3 Research Academy for Rural Revitalization of Zhejiang Province, Zhejiang A & F University, Hangzhou 311300, China

**Keywords:** multidimensional poverty, health, moderated mediation model, Jiangxi province

## Abstract

Although absolute poverty has been eliminated in rural China, multidimensional poverty has an unstoppable impact on the self-rated health of rural households through multiple dimensions. This study constructed a moderated mediation model with multidimensional poverty as the independent variable to explore the impact on rural households’ self-rated health, social capital as a mediating variable, and family care as a moderating variable. We used the survey data of 382 sample out-of-poverty rural households in Jiangxi, China, in 2020. Our results indicated that multidimensional poverty had a detrimental impact on the self-rated health and social capital of rural households, both of which were significant at the 1% level (β = −0.751, t = −4.775, and β = −0.197, t = −7.08). A test of the mediating effect of social capital using the mediation model found the mediating effect accounting for 84.95% of the entire effect of multidimensional poverty on rural households’ self-rated health. Further, the interaction term between family care and multidimensional poverty and its beneficial effect on social capital as well as the interaction term between family care and social capital and its negative effect on rural household’ self-rated health are both statistically significant at the 1% level (β = 0.558, t = −5.221 and β = −2.100, t = −3.304). It is revealed that multidimensional poverty affects rural households’ self-rated health through social capital and that family care moderates the mediating pathway. Family care exacerbates the negative effect of multidimensional poverty on rural households’ self-rated health and weakens the beneficial effect of social capital on rural households’ self-rated health. The lower (higher) the level of family care, the more significant the positive (negative) effect of social capital on rural households’ health. Therefore, rural households should prioritize building social capital and shifting the responsibility for family care. First, through enhancing housing infrastructure and establishing cultural and educational initiatives, households can improve their viability. Second, increasing engagement in group activities will enhance social networks and boost interpersonal connections. Finally, to lessen the stress on family caregivers, building socialized care services can cover the gap in family care.

## 1. Introduction

Although poverty affects all groups, it disproportionately affects those whose access to coping resources is most limited, such as rural households [1]. It is not limited to any single geographical region, income level, or age group [2]. Poverty is higher in rural areas than urban areas, and higher in non-metropolitan areas than in metropolitan areas. According to the Global Donor Platform for Rural Development, rural poverty must be tackled in order to meet the Millennium Development Goal because “three-quarters of the poor live in rural areas of developing countries.” [3]. Rural people in general were most affected by chronic poverty, and many rural households in emerging nations experience poverty even during prosperous years. Rural households are more likely to experience physical and psychological health issues due to poverty. Poverty has an adverse effect on people’s access to commodities and the quality of services, which has a bad effect on their health, according to the material pathway. In the psychological pathway, psychological stress (depression or pressure) may lead to poor consequences on mental health or physical health through behavior (smoking or alcohol abuse). Since the 1950s, sociological health research has routinely employed rural households’ assessments of various health outcomes for themselves as a typical indicator of health [4]. In China, as in the rest of the world, rural households have borne the brunt of poverty’s effects [5]. There are still large numbers of poor rural households in China and suffering from illness is the biggest cause of individual or temporary poverty in rural China today, while a lack of natural endowments, poor geography, and a fragile ecology are the main drivers of persistent poverty [6]. The term “poor families” refers to rural households classified as such by the Chinese government’s card-building procedure from 2020. In 2011, China’s rural poverty income benchmark was CNY 2300, and in 2020, it was CNY 4000. In 2020, absolute poverty was defined as having a net annual income per capita of less than CNY 627, while relative poverty was defined as having a net annual income per capita of CNY 865.

Although poverty was found everywhere and among all groups in China, it disproportionately puts certain groups at greater risk for well-being. After the implementation of large-scale poverty alleviation [7] and development, such phenomena have been alleviated and great achievements have been made [8]. From 1986 to 1993, China carried out large-scale developmental poverty alleviation, such as establishing special poverty alleviation work units and allocating special funds. During the 1994–2000 poverty alleviation period, the “8–7” poverty alleviation program was implemented, with the goal of solving the basic food and clothing problem of 80,000 rural poor people in about seven years; since the beginning of the current century, China has issued two new rural poverty alleviation and development programs; and in 2013, Chinese President Xi Jinping proposed the Targeted Poverty Alleviation (TPA) strategy [9]. The statistics from *Fifth Series of Reports on the Achievements of Economic* and *Social Development in the 40 Years of Reform and Opening Up* showed that the result was a dramatic decline in poverty rates, from 97.5% in 1978 to 0.6% in 2019. In 2020, the incidence of poverty was 0%. Currently, 98.99 million rural Chinese people who were living in poverty have been helped out of it. The complete victory in the fight against poverty marks the elimination of absolute poverty [10] based on income and the beginning of the transition to multidimensional poverty [11]. Absolute poverty is also called subsistence poverty. It is the inability of individuals and families to maintain their basic subsistence needs by relying on the income they earn from their labor and other legitimate income under certain social production and lifestyle patterns. As indicated, absolute poverty has been eliminated in China; there is no need to indulge on it. We focus on the concept of multidimensional poverty which somehow goes beyond the definitions of absolute and relative poverty and is a much more appropriate instrument to holistically measure poverty.

Contrary to the widespread use of absolute poverty, there is no single global standard for measuring relative poverty. When poverty was first defined, it meant not having enough money to cover the bare necessities of existence [12]. However, as socio-economic development has progressed, academics have continued to study poverty in new ways and comprehend it from perspectives other than just income [13]. Absolute poverty refers to subsistence poverty, which focuses on the basic survival and livelihood of individuals. Relative poverty refers to developmental poverty, which focuses on an individual’s ability to develop sustainably. The relative poverty theory proposed by Townsend (1979) [14] is a deepening and sublimation of the absolute poverty theory, which shifts scholars’ focus on poverty research from the basic survival needs of human beings to the inequality of income distribution and social deprivation, and puts more emphasis on all people sharing the benefits of economic and social development. Therefore, the understanding and assessment of poverty should encompass factors connected to an individual’s total development, such as health care, education, and standard of living, in addition to the single “income” dimension [15]. The “dual cut-off method” is the most commonly used method to create multidimensional poverty indexes [16]. By calculating the poverty line for each dimension, this method establishes an individual’s status as being below the poverty line in that dimension. Individuals who fall below the specified threshold for poverty in any one or more of the dimensions are then considered to be poor. This paper examines multidimensional poverty.

Poverty increases the risk of low health through poor environment, nutrition, and risky behaviors such as smoking, alcohol, and other things [17]. However, the relationship between poverty and rural households’ self-rated health is complicated by the vicious circle of poverty, unhealthy, and low income [18]. Self-rated health is a valid proxy for an individual’s health status because it is a subjective metric that depends on an individual’s nuanced appraisal of his or her own entirety [17,19,20]. Poverty hinders the improvement of rural household’ self-rated health; for example, it has a negative impact on the health level [21].

In addition, according to previous research, social relationships, networks, trust, and conventions are thought to form a relatively stable and institutionalized network known as social capital, which can improve social efficiency through cooperation. Social capital is a type of resource that helps members of the social structure live more comfortably or obtain financial advantages [22]. As for the rural households, social capital was considered to plays a mediating role between poverty and health [23], which can effectively alleviate the low health level caused by poverty [4]. First, social capital raises the human capital level and reward of rural households by increasing the probability of employment and income levels, health care expenditures, socioeconomic status, and borrowing capacity, thus reducing the probability of falling into poverty and avoiding a return to poverty [24]. Second, as a (semi-)acquaintance society, social capital plays a prominent influence in rural China, which helps rural households access health resources and has a significant positive effect on the health level of out-of-poverty households [25]. Therefore, social capital can reduce the occurrence of poverty and fundamentally avoid the low health level caused by poverty [26]. In addition, social capital can form a social environment of beneficial social interactions and mutual trust, which helps to obtain health information and effectively reduce health risks, thus affecting the level of health positively.

The impact of family care on mental health is a crucial area of current research. The more positive the family caregiving behavior for the care recipient, the higher the health index [27]. Meanwhile, family care can improve the physical and mental health, life happiness, and sense of accomplishment of caregivers [28]. Through reciprocal interactions, family care for the elderly and the children can result in a two-way exchange of resources, promoting the family’s social capital, the physical and emotional health of the individuals being cared for [4]. According to the *Role Enhancement* theory [29], providing care for a family increases social and familial support, emotional fulfillment, and caregivers’ ability to lead healthier lifestyles [30]. Research has also shown that providing care for family members might be detrimental to their physical and mental well-being [31]. Given the disparities between urban and rural areas, urban caregivers have better physical and mental health than rural caregivers [32]. According to the *Role Strain* idea [33], family caregivers may experience stress as a result of their duties, and high-intensity family care may take a toll on the health of caregivers [34]. This study broadens the definition of family care to include both childcare and elder care, in contrast to prior studies [24]. The care given by rural households to school-age children under the age of 16 is referred to as “child care,” while the care given to seniors over the age of 60 who need assistive technology to do daily tasks is referred to as “elder care.” Family care may operate as a moderator, even if the current findings have shown how social capital affects rural household health.

Thus, the objective of the research is to analyze the impact of multidimensional poverty on rural households’ self-rated health through theoretical modeling of rural households’ self-rated health. Since the independent variable in this paper is multidimensional poverty, it determines whether a farmer is in developmental poverty from all aspects of his or her life. The social capital is also the developmental capital formed from the trust, participation, support, and network established between the rural household and the outside world, which can influence the level of multidimensional poverty. Family care is a very common behavior in rural households, and through the care of the elderly and children can achieve a resource swap that affects the social capital of the caregiver by providing resources or taking up the opportunity cost of the caregiver. Meanwhile this study empirically tests the conclusions obtained from the theoretical model based on the survey data of 382 rural households lifted out of poverty in Jiangxi Province, China, in 2020. Our findings have important practical implications for the health and sustainability of rural households.

Previous literature discussing globally relevant documents on research topics has focused on the factors that influence multidimensional poverty. Battiston et al. (2013) [35] have analyzed multidimensional poverty indices for six Latin American countries, including Argentina, Brazil, Chile, and so on, over the period 1992–2006 and found that a lack of adequate sanitation and education for household heads were the biggest factors in overall multidimensional poverty. Pinilla-Roncancio et al. (2020) [36] compared the multidimensional poverty levels of people with and without disabilities in Guatemala, Cameroon, and India, with health-related indicators having the greatest impact on the multidimensional poverty levels of people with disabilities. Fransman and Yu (2019) [37] have calculated an annual multidimensional poverty index for South Africa, which shows that Africans experience more than 95% of multidimensional poverty and that unemployment, years of schooling, and disability are the three indicators that cause the most poverty. Martinez and Perales (2017) [38] have examined how year-to-year changes in multidimensional poverty in Australia in recent years have been driven primarily by fluctuations in social support, health, and material resources, suggesting that the effectiveness and efficiency of poverty reduction policies should be improved by focusing on improving disadvantage in the areas of health and material resources. Based on different measurement methods, Padda and Hameed (2018) [39] estimated the level of multidimensional deprivation and poverty in rural Pakistan through socio-economic scores and found that 44% of rural households endured sustainable deprivation and well-being issues, particularly in the areas of health, stunting, the standard of living, and so on. Based on the Colombian Multidimensional Poverty Index (CMPI), Angulo et al. (2016) [40] have concluded that multidimensional poverty in rural Colombia decreased between 1997 and 2010, but imbalances remain. It can be seen that multidimensional poverty has received widespread attention at the international level. In practice, the European Union (EU) has adopted a multidimensional poverty and social exclusion target for 2020, justified by the fact that the calculation of a multidimensional poverty index is an effective way of communicating in a political context and a necessary tool for monitoring the situation in different countries [41].

Compared with the existing literature, the possible marginal contributions of this paper are mainly manifested in the following basic aspects. First, in the context of absolute poverty alleviation, we use the MPI (Multidimensional Poverty Index) [42] mentioned in the Sustainability Development Goals (SDGs) guidelines to select multidimensional indicators that can cover all aspects of life to judge rural households who are in relative poverty [43]. To ascertain if rural households are multidimensionally poor, the income indicator is also used as a separate dimension. This study develops a comprehensive income-based multidimensional poverty identification and assessment method. Second, previous research has looked at the impact of social capital and multidimensional poverty on rural households’ health separately, with the majority of the literature focusing on just one aspect of social capital. In order to provide a more accurate and robust estimate of rural households’ social capital, the impact of social capital is examined in this study by evaluating the entire social capital index of farmers across four dimensions: social networks, social trust, social participation, and social support. We investigate the impact of multidimensional poverty on rural households’ self-rated health, and further investigate the mitigating or facilitating effect of entropy-valued social capital on the self-rated health of rural households affected by multidimensional poverty based on Bootstrap [44], which can be reliably obtained by running Bootstrap independent sampling 5000 times. Third, we introduce family care into the empirical model and examine the moderating effect of family care on the mediating effect based on Bootstrap. Rural households regularly engage in family care, but less research has been conducted on the factors affecting the health of rural households in light of this widespread life behavior. We investigate whether multidimensional poverty has an impact on rural households’ health at the micro level and study its mechanisms of influence to determine whether low-intensity family care and high-intensity social capital can be a significant opportunity to improve the health of rural households. Thus, our study on multidimensional poverty and rural household’ health has both significant theoretical and real-world implications.

The rest of the paper is organized as follows. Section 2 presents the theoretical hypothesis of this paper based on relevant theories and existing studies. Section 3 introduces the data sources and the basic setup of the model and analyzes the moderating or facilitating role of social capital in the impact of multidimensional poverty on rural households’ self-rated health and the moderating role of family care in the framework of this theoretical model. Section 4 conducts an empirical analysis to verify the conclusions obtained from the theoretical model based on the survey data from Jiangxi Province, China. Finally, we draw discussions and conclusions in Section 5.

## 2. Analytical Framework and Assumptions

### 2.1. The Impact of Multidimensional Poverty and Health

**Figure 1:**
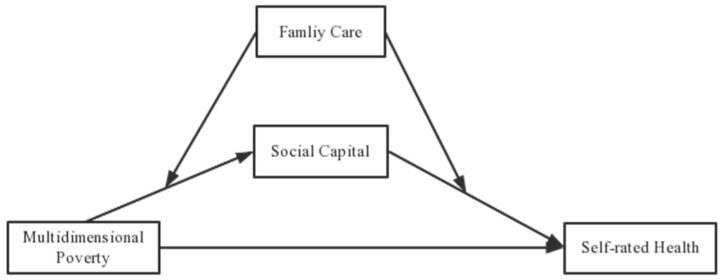
Hypothetical model diagram of the mediating role of social capital and the moderating role of family care.

Figure 1 is the hypothetical model diagram of the mediating role of social capital and the moderating role of family care. In contrast to the conventional use of “poor income” as the criterion for defining poverty, Sen has presented the *Feasible Capacity* theory, asserting that the key notion of the poverty theory is “feasible capacity”. Sen defines poverty, based on the capabilities approach, as a deprivation of capabilities and as a lack of multiple freedoms people value and have reason to value [45]. Sen’s feasible capacity theory, which defines the poverty concept from a philosophy perspective, transcends a single subject such as economics, sociology, politics, and so on, and builds the basis of multidimensional poverty theory [46]. According to the theory, realizable capacity can actualize a large variety of functional activity combinations. The feasible capacity theory stresses the numerous facets of improving people’s lives, emphasizing that poverty is determined by a variety of characteristics, including education, access to healthcare, and basic daily needs, and that it is not just an issue of income [47]. It is logical to suppose that poverty affects not just one area of one’s health but a number of different dimensions as well. The feasibility capacity theory-based design of a multidimensional poverty measure allows for investigation of the factors that prevent people from experiencing the pleasure of excellent health [48]. Lack of access to preventative healthcare services makes people living in many forms of poverty more vulnerable to malnutrition and dangerous living conditions, both of which may have an adverse effect on their health [49].

In this study, rural households’ self-rated health status served as a proxy for their general health. The multidimensional poverty level of rural households was assessed using health, education, living standard, and income [50]. Based on this, this study proposes Hypothesis 1 (H1):

**Hypothesis 1 (H1).**  *Multidimensional poverty may negatively affect the rural households’ self-rated health.*

### 2.2. The Mediating Influence of Social Capital on the Relationship between Multidimensional Poverty and Self-Rated Health

Social capital, which enables people to cooperate to increase social advantages, includes social networks, social trust, and social norms [51]. Social networks provide an essential form of social support called social capital, from which individuals can benefit [52]. It can assist people in locating reliable information, financial, emotional, and spiritual assistance [53]. The social identity, social trust, and sense of belonging that people have developed through social networks can successfully mitigate the negative effects of multidimensional poverty on physical and mental health in many dimensions of life [54]. It is also feasible to expand knowledge about health issues, promote health consciousness, and increase the variety of opportunities for engaging in healthy activities by developing strong social connections with other people [55].

In this study, the total social capital index of rural households was calculated using four dimensions: social networks, social trust, social participation, and social support [56]. Based on this, this study proposes Hypothesis 2 (H2):

**Hypothesis 2 (H2).**  *Social capital may play a mediating role in the way multidimensional poverty affects rural households’ self-rated health.*

### 2.3. The Moderating Effect of Family Care

Family care, which largely refers to the loving care supplied by parents to their children and the supportive care delivered by children to their parents, is the main type of care for older people with disabilities and children in Chinese families [57]. A variety of social structural changes, including the miniaturization of China’s family structure and the change in women’s employment status, have made it harder for people to support their families [58]. Because they feel their futures will be shorter as they become older, people prioritize preserving and improving social interactions with their close friends and families, according to *Socioemotional Selectivity Theory* [59]. By offering support, low-intensity family care may help enhance the mental and emotional health of caregivers who provide other family member with care in households. However, high-intensity family care requires more opportunity costs for caregivers, taking up more of the family’s social capital and the caregiver’s work or leisure time. Family carers’ physical and emotional health suffers as a result of intense caregiving. Additionally, this procedure may slow the development of caregiver’s social capital [32]. Both elder care and childcare need be taken into account when researching family care. Caregiving for children and elderly has varied effects on the health of the caregivers [60]. In childcare, educational care is necessary in addition to everyday, emotional, and physical care for the kids. However, the psychological strain of looking after an aging parent can be harder because there are not as many “expectations” as there are with caring for young children [61].

In this study, the number of children and elderly care recipients (school-age children under the age of 16 and elderly over the age of 60 who need assistive technology to do daily tasks) was counted in order to assess the level of family care in rural household [59]. Based on this, this study proposes Hypothesis 3 (H3):

**Hypothesis 3 (H3).**  *Family care may moderate the mediating pathway multidimensional poverty-social capital-self-rated health.*

## 3. Materials and Method

### 3.1. Data Sources and Sample Selection

Jiangxi Province is a well-known historical province and one of China’s key areas for fighting poverty. As a major grain exporting province Jiangxi Province is also an economically underdeveloped province in the central region. It has disadvantages in terms of human capital, infrastructure, and geographic location [62]. The province has 58 former Central Soviet counties, 17 Luoxiao Mountain Area counties, 21 key counties for poverty alleviation and development, 25 poor counties, and 3058 poor villages in the “13th Five-Year Plan”, including 269 deep-poverty villages.

Jiangxi Province is the focus of our investigation for three reasons. First of all, non-poor households in this area have exceptional social capital and multidimensional relative poverty status. An important priority area for the eradication of poverty is a historic revolutionary area, which makes up more than 80% of the province’s land area. Second, the social capital of households in the area is representative. The government offers poor households substantial help in terms of output and means of subsistence. The history of the former revolutionary area also provides the visiting sites with historical and cultural significance to the ruling Communist Party’s history resources. Third, among other terrains, the five counties in the study region include plains, hills, and mountains. They span a vast array of topographical features and geographic areas. Fourth, Jiangxi Province helped 3.46 million people out of poverty in 2020, achieving region-wide poverty alleviation. Out-of-poverty households’ per capita income climbed from CNY 3344 in 2015 to CNY 12,626 in 2020 [51]. It can be seen that Jiangxi Province has achieved a wide range of poverty alleviation results. Additionally, the “No. 1 central document” for 2022 published by the Chinese government explicitly states the need to prevent households that have been lifted out of poverty from returning to poverty, despite the fact that rural households in the region have been fully lifted out of poverty and are no longer at risk of doing so. One of the most vulnerable groups in China in terms of sustainable lives is rural households that have managed to escape poverty; therefore, our concern about this group is crucial for building on China’s recent progress in reducing poverty. Consequently, the survey sample in this area is comprehensive and can accurately represent various topographic features and levels of poverty.

Jiangxi Province’s recorded number of poor people dropped from 3.46 million in 2013 to 96,000 at the end of 2019. Poverty prevalence fell from 9.21% to 0.27%. According to the 13th Five-Year Plan, all 3058 of the province’s impoverished villages would be out of poverty by 2020. The per capita disposable income of households out of poverty increased from CNY 4102 in 2015 to CNY 14,452 in 2021.

We conducted a field survey in Anyi County, Jinxian County, Nanchang County, Wanli District, and Xinjian District in Jiangxi Province in the summer of 2020 to gather data for this research. Households were selected using a stratified random sampling method. Five counties were identified based on the endowment of land resources and rural household’s income; then, 8 villages were chosen in each county, and 10 out-of-poverty households were randomly selected in each village. Thus, the sample contains 400 rural households from 40 villages in 5 counties.

Questionnaires were developed and were administered by Masters and Ph.D students as well as the teachers from our research team at Jiangxi Agricultural University. A week before the investigation, the teacher in charge of the project trained the interviewers for five days on the content of the questionnaire. A total of 400 questionnaires were collected through face-to-face interviews between the trained interviewers from the research group and the head of the rural households or another knowledgeable adult during the research process. After removing invalid questionnaires that omitted important information and were inconsistent, we collected 382 valid questionnaires, with a sample validity of 95.5%. The following three categories are included in the questionnaire’s content: (1) rural households demographics (including age, gender, education, health, income, and marital status, etc.); (2) household capital (including social capital, human capital, furniture capital, and housing capital, etc.); (3) household livelihood strategy (including energy use, income sources, consumption sources, agricultural operations, and non-agricultural operations, etc.).

During the study in 2020, the study area was hardly affected by COVID-19, and there were no confirmed cases until nearly three months before the filed survey. On the other hand, the impact of COVID-19 on rural households’ livelihoods was also investigated in our study, but most farmers responded that the impact was not significant. Thus, the study did not analyze the health impact caused by COVID-19 because the sample was limited to Jiangxi and there was a lack of reference samples from other regions.

### 3.2. Variable Selection

#### 3.2.1. Dependent Variable

Health self-evaluation has been widely used in the literature as a comprehensive indicator that is simple to gather and of good quality [63]. In order to assess the rural households’ health, this study used the self-rated health as the dependent variable. Despite the extremely subjective nature of rural households’ self-evaluations, their replies reflect objective health indices such as individual mortality and major diseases [64]. According to the five categories that respondents used for their answers—“very poor, poor, average, good, very good”—dependent variable values were assigned from 1 to 5.

#### 3.2.2. Independent Variable

Multidimensional poverty serves as the study’s independent variable. The choice of dimensions and associated indicators for multidimensional poverty is flexible. The appropriate dimensions must be selected to gauge local poverty in different climes, with different cultural characteristics and different consumer habits [65]. The MPI, which is included in the Sustainability Development Goals (SDGs) recommendations, served as the study’s foundation [66]. The MPI contained a reasonably developed and valuable index system that could indicate people’s fundamental viability [67]. The indicators used in this study were chosen in accordance with the Global MPI analytical framework and were based on the customs of the research locations, the specificity of the research subjects, and the availability of data, combined with China’s poverty alleviation policies and goals, developed different threshold values for the indicators, and appropriate adjustments were made to the MPI model for China [43,68]. This was performed by combining the existing research bases worldwide, such as Callander [69], Pasha [70], and Zhang [71]. The MPI’s three current dimensions—health, education, and living standard—were initially used as a starting point for the study’s dimension selection. However, the income indicator was later included as a separate dimension. This article makes the case that income, education, access to healthcare, and living conditions should all be considered when choosing relative poverty metrics. Education includes 2 indicators: years of education and children’s enrollment. Health includes 2 indicators: medical expenses and health insurance. Living Standard includes 5 indicators: electricity, water, floor, cooking fuel, and assets. Income includes 1 indicator: per capita disposable income. This means there are 10 indicators in total [72]. First, in the framework of China’s poverty alleviation policy and taking into account the endogeneity issue, the indicators of the health dimension were restricted to medical treatment. Second, the cost of education is typically the main factor contributing to the low enrollment rates of children in rural areas, and the education indicator is constrained by data restrictions and the fact that this cost encompasses learning materials, lodging fees, and travel expenses between regions [71]. The enrollment rate of kids in school-age has surpassed 99.94% since the introduction of obligatory nine-year education in 2006 (Data source: 2019 National Educational Development Statistical Bulletin.) We substitute the relative educational disadvantage indicator for the indicator of the enrollment rate of youngsters. As a measure of relative educational disadvantage, we employ the widely established academia poverty line judgment criterion. Whether rural households devote more or less than 40% of their median per capita disposable income to their children’s education is how we measure relative educational disadvantage. Finally, living standards are expressed using five indicators depending on the data that is available: electricity, cooking fuel, floor, assets, and per capita housing area. Accordingly, the selected dimensions and indicators are as follows:

(1) Health

For health, we used two indicators: medical expenses and health insurance. A member of the household who incurs medical costs due to a major illness or hospitalization is regarded to be lacking the medical expenditure indicator [73]. Likewise, a member of the household who lacks medical insurance in rural areas is deemed to be lacking the medical insurance indicator [58]. Since China has recently placed a strong emphasis on health insurance coverage, the choice of health insurance can gauge the government’s success in reducing poverty in this area as well as the efficacy of rural households’ participation [74]. In China, the reimbursement rate for rural health insurance for the average rural resident is 60% for town health centers, 40% for secondary hospitals, and 30% for tertiary hospitals. The benefit for poor households was that they were entitled to government-subsidized rural health insurance subscriptions, with specific reimbursement rates determined by each province and city. For example, in Jiangxi province, full subsidies have been given to special hardship cases to participate in the insurance, and the starting line for major medical insurance was reduced by 50% for special hardship cases, low-income recipients and those returning to poverty, with no annual maximum payment limit.

(2) Education

For education, we used two indicators: years of education and child enrollment. The education years indicator is considered deprived for household members over 16 years old who have less than 9 years of education. The child enrollment indicator is regarded to be insufficient if enrollment costs for children under the age of 16 in a household exceed 40% of the national median per capita disposable income for rural inhabitants in 2019 (CNY 5755.6) [75]. Despite the fact that China has been implementing a nine-year compulsory education, pupils still need to attend school in different locations due to the lack of schools in rural areas, particularly in remote areas, which may result in additional costs such as school boarding.

(3) Living Standard

The standard of living dimension comprises five indicators: electricity, cooking fuel, floor, assets, and per capita housing area [70]. The indication of electricity is said to be deprived if the family is without electricity. The cooking fuel indicator is deprived if the primary fuel for cooking is unclean fuel, such as firewood. The floor indicator is deemed to be deprived if the structure is made of mud. The assets indicator is deemed to be deprived if households do not own more than one of the following assets: battery car, car, television, refrigerator, washing machine, telephone, air conditioner, computer, electric heater, or water heater. Less than 12 square meters are seen as a sign of deprivation in terms of housing area per capita.

There is no consensus in the current literature on how to select indicator weights and most current Eastern and Western poverty researchers have generally adopted the equal weighting approach. Some studies have concluded that different weight setting techniques have no significant influence on the multidimensional poverty measurement result [65]. Given that the relevance of each indicator in evaluating rural household survival is not considerably different and that the equal-weight approach makes the final multidimensional poverty index measurement results similar, drawing on the Global MPI analysis framework, this article uses the equal-weight method (equal weights of dimensions and indicators). The weights are nominally assigned to each dimension, to constitute an index with equally weighted dimensions, that is one third each. This paper analyzes 10 indicators in 4 dimensions: health, education, living standard, and income. Indicators that do not reach the threshold value are assigned a value of 1, indicating that the monitored households are in poverty [43]. The weight of each dimension is 1/4, and the indicators within each dimension are equally distributed according to the number of indicators, 1/4, 1/8, and 1/20, respectively [76]. The annual total value of each dimension index of monitoring farmers exceeds 1/3 (0.33), to be considered multidimensional poverty as per the MPI [71]. The global MPI considers individuals to be vulnerable to multidimensional poverty if they are deprived in a weighted indicator between 20% and 33.33% (close to the one-third threshold). The comprehensive information on the threshold value and weight setting of each index is shown in Table 1.

(4) Income

For income, we use one indicator: per capita disposable income. Income can reflect the poverty level of rural households to a large extent. The threshold value for rural households’ income in this paper was determined as a percentage of the median disposable income of rural inhabitants, which was set at 40% of the median per capita disposable income of rural residents nationwide [77], rather than using the absolute poverty line as the standard. Per capita disposable income of rural households below 40% of the 2019 national median disposable income per rural resident (CNY 5755.6) is considered deprived of the per capita disposable income indicator.

#### 3.2.3. Mediating Variable

This study uses social capital as a mediating variable and picks four dimensions from the existing literature for measurement: social networks, social trust, social participation, and social support [56]. The entropy value of social capital is calculated by these four dimensions, and the total social capital index is measured.

(1) Social networks that link people from various backgrounds and give them the chance to trade fresh knowledge or resources across various contexts are often referred to as “bridging social capital” [78]. According to Knoke, social networks are structures made up of a number of organizers in which some of the participants are linked by one or more relationships [79]. Social networks give access to various people to a variety of resources, including knowledge, morals, and financial resources. Through their intimate relationships with people from various backgrounds, individuals can be able to access social and/or emotional support that would otherwise be impossible if they simply relied on their networks of family and friends [78].

Rural China is a society of human relationships, especially a society of acquaintances where blood ties, etc. are an important components of rural households’ social capital. Accordingly, this paper chose the question “How many relatives in your family are village, township cadres or other public officials” in the questionnaires as a proxy variable for the social networks, drawing on the research of Daley [80].

(2) A mutually accepted expectation of social trust produces solid and reliable connections between people and their environment [81]. These interactions allow participants to covertly rely on one another for a variety of requirements. The benefits of trust, such as cooperation and exchange, are promoted by peoples’ trust in public institutions such as the government. It is simpler to cooperate to achieve shared objectives the more trust there is within society [82]. Accordingly, this study chose the question “Do you trust the government?” as a proxy variable for social trust, and the response was separated into “very trusting, trusting, general, distrustful, very distrustful” each with a value of 1–5.
(3) Rural households foster engagement, cooperation, and mutual aid among neighbors through participating in social activities that improve their ability to communicate with others. This paper chose the question “Whether your family participates in organizations such as planting associations and cooperatives” as a proxy variable for social participation and gave a value of 1 to those who do and a value of 0 to those who do not, in accordance with the research of Lu [83].
(4) In rural China, the ability of neighbors to provide a hand to struggling rural households is a significant means of social contact and a manifestation of mutual support. Accordingly, this research chooses “How many people will come to help if the family holds weddings and funerals” as a proxy variable for social support, drawing on Canto’s study [84].

#### 3.2.4. Moderating Variable

The moderating variable in this study is family care. Family care in this study refers to both child and elder care. Childcare is defined as the care given to children under the age of 16 for living and education depending on their capacity to live independently. This definition is consistent with previous studies in the relevant field [61]. According to the definition of elderly care, it refers to senior adults above the age of 60 who need assistive technology to do daily tasks. In order to measure the number of people who require family care as a proportion of the total household size and to examine the intensity of family care, this paper chooses from the questionnaire “the number of children under 16 years old in the household” and “the number of elderly people over 60 years old in the household who need assistance in daily life”, referring to the studies of Minty [61].

#### 3.2.5. Control Variables

Numerous factors can affect rural household health, thus in order to reduce error, this study chose control variables from two levels: individual characteristics and household characteristics. The household head’s gender, age, education level, and marital status were chosen as individual characteristics; total household size, labor force participation rate, and total household income (log) were chosen as household characteristics [85]. Table 2 displays each variable’s precise definition.

#### 3.2.6. Descriptive Statistics

Table 2 shows the variable definitions and descriptive analysis for the total sample, and Table 3 shows the one-way ANOVA results for the multidimensional poverty sample and the non-multidimensional poverty sample. In Table 2, from the total descriptive analysis results of rural households, the average self-rated health of rural households was 2.510, which was low. The average level of multidimensional poverty among rural households was 0.350, meaning that 35% of them fall into multidimensional poverty. This statistic illustrates how significantly rural households were able to reduce poverty.

From the perspective of the social capital of rural households, the average value of the total social capital index of rural households was 0.225, which indicated that the average social capital of rural households was low. The average value of the social networks of farmers was 0.235, reflecting that there were fewer villages, township cadres, or other public officials in rural households, and the social networks were low. Rural households had a social trust rating of 3.298 on average, which indicated that they trusted the government more. The average level of social participation among rural households was 0.397, which indicated that 39.7% of rural households belonged to groups such as cooperatives or planting societies. The mean value of social support for rural households was 14.505, reflecting that rural households had an average of 15 people to help with red and white celebrations. From the perspective of the level of family care for rural households, the proportion of children under 16 and elderly people over 60 in rural households was 45.8%. This meant that nearly half of the members of rural households need to be cared for.

From the perspective of personal characteristics of rural households, 82.1% of rural households were male, 82.1% of rural households were male, the majority of rural households were married, the average age of the rural households surveyed was 59.185, the average number of years of schooling was 4.229, and the average marital status was 2.583. They had a poor level of education, meanwhile, and there was a general aging tendency among them.

From the perspective of family characteristics of rural households, the average number of rural households in the family was 2.850, and the average number of laborers was 0.926, reflecting that the number of laborers in a family was less than half of the total. The log of total household income was 8.831.

The one-way ANOVA findings were presented in Table 3 and were divided into two categories: multidimensional poverty and non-multidimensional poverty. The independent sample T-test in SPSS14.0 was used for analysis, and it was discovered that the mean value of self-rated health for rural households experiencing multidimensional poverty was 1.990, which was lower than the mean value for rural households experiencing non-multidimensional poverty, which was 2.790, and was significant at the 1% level. The mean values of the total social capital index (0.099), social networks (0.040), social trust (2.890), and social participation (0.358) for rural households with multidimensional poverty were lower than the mean values of the total social capital index (0.254), social networks (0.340), social trust (3.520), and social participation (0.500) for rural households with non-multidimensional poverty, and these differences were significant at the 1%, 1%, and 5% levels, respectively. The mean value of years of education for rural households experiencing multidimensional poverty was 3.570, which was lower than the mean value for rural households experiencing non-multidimensional poverty, which was 4.585, and was significant at the 5% level. The mean total household size for rural households experiencing multidimensional poverty was 3.130, which was greater than the mean total household size for rural households experiencing non-multidimensional poverty, which was 2.700, and was significant at the 10% level. Rural families with multidimensional poverty had a mean total household labor force of 1.100, which was more than that of rural households without multidimensional poverty by 0.830 and statistically significant at the 10% level. The mean total household income for rural households experiencing multidimensional poverty was 6.491, which was significantly lower than the mean total household income for rural households experiencing non-multidimensional poverty, which was 10.105, and statistically significant at the 1% level.

### 3.3. Methods

#### 3.3.1. The A-F Multidimensional Poverty Methodology

The measure of multidimensional poverty in this paper was based on the “dual cut-off method” (A-F method) proposed by Alkire and Foster [66]. The method identified poverty through a dual cut-off. The first step was defining the set of dimensions that will be considered in the multidimensional measure. The second step was setting a dual cut-off for judging the poverty of the sample. The first cutoff was setting the deprivation cut-offs for each dimension, applying the cutoffs to ascertain whether each person was deprived or not in each dimension. The second cutoff was determining the poverty cutoff, therefore identifying individuals with a dimensional poverty measure above a certain cutoff as poor and identifying each person as multidimensional poverty or not according to the selected poverty cutoff.

(1) Identification of one-dimensional poverty

Let *n* represent the individuals and let *d* represent the number of indicators under analysis. Then, the n*d dimensional sample observation matrix is obtained, *X* = [*x_ij_*] is the achievement of individual *i* in indicator *j*, i.e., x*_ij_* ∈ R, (*i* = 1,2,..., *n*; *j* = 1, 2,..., *d*):

(1) $$X=\left(\begin{array}{cccc} x_{11} & x_{12} & \cdots & x_{1d}\\ x_{21} & x_{22} & \cdots & x_{2d}\\ \vdots & \vdots & \ddots & \vdots \\ x_{n1} & x_{n2} & \cdots & x_{nd} \end{array}\right)$$

For each indicator *j*, a deprivation cutoff *z_j_* is set, and a respondent is considered to be deprived on a dimension if its well-being value falls below that cutoff (as shown in Table 1). From this, *Z* can be defined as the row vector that collects the deprivation cutoffs and a n*d matrix of deprivation *G* = [*g_ij_*] is obtained, *g_ij_* represents the deprivation status of individual *i* on index *j*:

(2) $$G=\left(\begin{array}{cccc} g_{11} & g_{12} & \cdots & g_{1d}\\ g_{21} & g_{22} & \cdots & g_{2d}\\ \vdots & \vdots & \ddots & \vdots \\ g_{n1} & g_{n2} & \cdots & g_{nd} \end{array}\right)$$

If *x_ij_* < *z_j_*, then *g_ij_* = 1, indicating that individual is deprived in the indicator; if *x_ij_* ≥ *z_j_*, then *g_ij_* = 0, indicating that individual *i* is not deprived in the indicator, that is:

(3) $$g_{ij}=\left\{\begin{array}{cc} 1, & x_{ij}<z_{j}\\ 0, & x_{ij}\geq z_{j} \end{array}\right.$$

(2) Identification of multidimensional poverty

This paper adopts the dimension equal weight method. Let *w_j_* be the vector of weights that reveals the equal importance of each indicator *j*, then $\sum_{j=1}^{d}w_{j}=1$. To judge whether a rural household is in multidimensional poverty, multiple dimensions of poverty are summed to obtain the multidimensional poverty index (*MP_i_*) for the individual *i*:

(4) $$MP_{i}=\sum_{j=1}^{d}w_{j}*g_{ij}$$

This paper draws on Pasha [55]. Let *k* = 1/3 denote the poverty cutoff. The poverty cutoff is implemented by using the method of identification *p_i_*, which identifies individual *i* as multidimensional poverty when their deprivation score is at least *k*. That is *MP_i_* ≥ *k*, otherwise non-multidimensional poverty:

(5) $$p_{i}=\left\{\begin{array}{cc} 1 & MP_{i}\geq k\\ 0 & MP_{i}<k \end{array}\right.$$

Next, the number of multidimensional poor households is identified, and the multidimensional deprivation matrix Q:

(6) $$P=\left(\begin{array}{cccc} p_{11}(1) & p_{12}(2) & \cdots & p_{1d}(d)\\ p_{21}(1) & p_{22}(2) & \cdots & p_{2d}(d)\\ \vdots & \vdots & \ddots & \vdots \\ p_{n1}(1) & p_{n2}(2) & \cdots & p_{nd}(d) \end{array}\right)$$

(3) Multi-dimensional poverty index calculation

First, computing the multidimensional headcount ratio or the incidence of multidimensional poverty: *H*, that is, the ratio of individuals identified as multidimensional poverty among *i* individuals. Second, computing the average share of weighted indicators in which poor people are deprived. This entails adding up the deprivation scores of the poor and dividing them by the total number of poor people. This is the intensity of multidimensional poverty, *A*. Third, computing the multidimensional poverty Index *M*, *M* = *H* × *A*, that is, the multidimensional poverty index is equal to the product of the two previous partial indices. The number of multi-dimensional poor people is represented by *q*, and the formula is as follows:

(7) $$H=\frac{\sum_{i=1}^{n}p_{i}}{n}$$

(8) $$A=\frac{1}{q}\sum_{i=1}^{q}p_{i}$$

(9) M=HA

(4) Dimensional decomposition

Further, the multidimensional poverty index is decomposed by indicator, and *M_j_* is the contribution of the dimension to the multidimensional relative poverty index, and *q_j_* is the total number of multidimensional relatively poor individuals who are deprived in the dimension. The contribution rate of each indicator to the multidimensional poverty index is:

(10) Ij=qjNwjM

#### 3.3.2. Multivariate Ordered Logistic Model

This paper selected the Bootstrap-based mediation effect test model and the moderated mediation effect test model proposed by Preacher and Hayes. This paper explored the impact of multidimensional poverty on the rural households’ self-rated health. The study was conducted in two steps.

The first step, based on Bootstrap’s mediation effect test model, was selected to explore the impact of multidimensional poverty and social capital on the rural households’ self-rated health. The setup model was as follows:

(11) $$Y_{i,t}=c+\alpha_{1}X_{i,t}+\sum Control_{i,t}+\varepsilon_{i,t}$$

(12) $$M_{i,t}=c+\alpha_{2}X_{i,t}+\sum Control_{i,t}+\varepsilon_{i,t}$$

(13) $$Y_{i,t}=c+\alpha_{3}X_{i,t}+\beta_{1}M_{i,t}+\sum Control_{i,t}+\varepsilon_{i,t}$$

Among them, *X*_*i*,*t*_ represents the multidimensional poverty status of rural households, *M*_*i*,*t*_ represents the social capital level of rural households, *Y*_*i*,*t*_ indicates the rural households’ self-rated health, *α* indicates the coefficient vector group, and *ε* indicates the random error term. Next, the robustness of the model was verified. In the econometric analysis, robust standard errors were used to eliminate the influence of heteroscedasticity on the model results.

The second step is to test Hypothesis 3, a Moderated Mediation Model (MMM) is further constructed using family care as the moderating variable as follows:

(14) $$Y_{i,t}=c+\chi_{1}X_{i,t}+\chi_{2}M_{i,t}+\chi_{3}X_{i,t}\times W_{i,t}+\chi_{4}M_{i,t}\times W_{i,t}+\sum Control_{i,t}+\varepsilon_{i,t}$$

where *W*_*i*,*t*_ stands for the degree of family care, *X* × *W* for the phrase that describes the relationship between multidimensional poverty and family care, and *M* × *W* for the term that describes the connection between social capital and family care. The above econometric models were all implemented by SPSS.

## 4. Results

### 4.1. Test for the Mediating Effect of Social Capital

The *Model 4* tested the mediating role of social capital in the relationship between multi-dimensional poverty and self-rated health in the SPSS macro created by Hayes [86] (*Model 4* is a simple mediation model), while controlling the individual characteristics, family characteristics, and income. In Table 4, *Models 1* and *2* illustrate how multidimensional poverty affects social capital and the self-rated health of rural households, respectively. With social capital serving as a mediating variable, *Model 3* illustrates the impact of multidimensional poverty on rural households’ self-rated health. The results in *Models 1* and *2* showed that multidimensional poverty had a detrimental impact on self-rated health and social capital, both of which were significant at the 1% level (β = −0.751, t = −4.775 and β = −0.197, and t = −7.08, respectively). Additionally, in *Model 3*, the direct detrimental effect of multidimensional poverty on self-rated health was not significant when the mediator variable was added, while the beneficial impact of social capital on self-rated health was significant at the 1% level (β = 3.240, t = 13.482).

Furthermore, the mediation effect was tested using the parameter Bootstrap. According to the regression results in Table 5, the middle of the Bootstrap 95% confidence interval for the mediating effect of social capital on multidimensional poverty on self-rated health did not contain 0, and the upper and lower limits of the interval for the direct effect of multidimensional poverty on self-rated health both contained 0. It demonstrated that social capital had a fully mediating effect, with the mediating effect accounting for 84.95% of the entire effect of multidimensional poverty on rural households’ self-rated health. The possible reason for this is that the choice of core variables in this paper is multidimensional poverty rather than single-dimensional poverty due to income. The causes of multidimensional poverty come from all aspects of the rural household’s life, which also reflect the level of social capital of the rural households. Therefore, a greater portion of the influence of rural households’ self-rated health is explained by the mediating effect of social capital. Both Hypothesis 1 and 2 were confirmed. *Model 1* tested Hypothesis 1 and demonstrated that multidimensional poverty reduced rural households’ self-rated health. *Model 3* tests Hypothesis 2 and demonstrates that social capital mitigates the effect of multidimensional poverty on farmers’ self-rated health.

### 4.2. Test for Mediating Effect with Moderation

The moderating influence of family care was examined while controlling for individual, family, and rural households income using *Model 58* in the SPSS macro created by Hayes (*Model 58* assumes that the front and back segments of the mediation model are subject to moderation, consistent with the theoretical model of this study) [86]. *Models 4* and *5* examined the moderating impact of family care on the pathway of multidimensional poverty affecting social capital and the pathway of social capital influencing farmers’ self-rated health, respectively. According to Table 6, the interaction term between family care and multidimensional poverty and its beneficial effect on social capital were both statistically significant at the 1% level (β = 0.558, t = −5.221). It demonstrated how family care had a moderating effect on the relationship between multidimensional poverty and social capital. It also demonstrated how multidimensional poverty negatively affected rural households’ social capital level. Rural households’ self-rated health was negatively impacted by the interaction term between family care and social capital at the 1% level (β = −2.100 and t = −3.304). Meanwhile it demonstrated that family care could moderate the relationship between multidimensional poverty and rural households’ self-rated health by weakening the positive effects of social capital on the rural household health. The effects of multidimensional poverty on social capital and the effects of social capital on self-rated health were both significantly mitigated by family care, thus hypothesis 3 was validated.

*Model 58* also examined the mediating effect with moderation. It is well known that the Bootstrap approach will produce high, medium, and low groups for the moderator variable by adding or deleting one standard deviation from the mean, and that the moderator variable’s significance will then be determined by the coefficient under the mediating effect. The judgment methods are as follows, according to the analysis of the results provided by Ye [87], if some of the mediating effects of the three groups were significant and some were not significant, it indicated that the mediation effect was significantly different from the moderating effect. The high-level family care group (M + 1SD), as shown in Table 7, at the three levels of family care, did not significantly differ from the other groups in terms of the mediating role of social capital in multidimensional poverty and rural households’ self-rated health (LLCI = −0.016, ULCI = 0.084). With 95% confidence intervals of [−0.670, −0.334] and [−0.219, −0.057], respectively, social capital had significant mediation effects on multidimensional poverty and rural households’ self-rated health in the low-level family care group (M-1SD) and the mean family care group (M). That is, by lowering the multidimensional poverty level of rural households, social capital is less likely to have an impact on the rural household health as the family care level of the tested family improves.

Family care was divided into high and low groups according to the mean plus or minus one standard deviation, and the effects of social capital on rural households’ self-rated health and the effects of multidimensional poverty on social capital at different levels of family care were analyzed using simple slopes.

**Figure 2:**
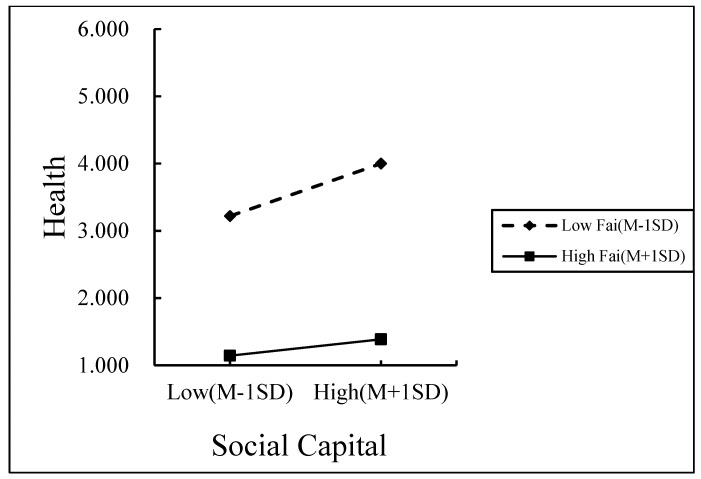
The moderating role of family care in the relationship between social capital and health.

**Figure 3:**
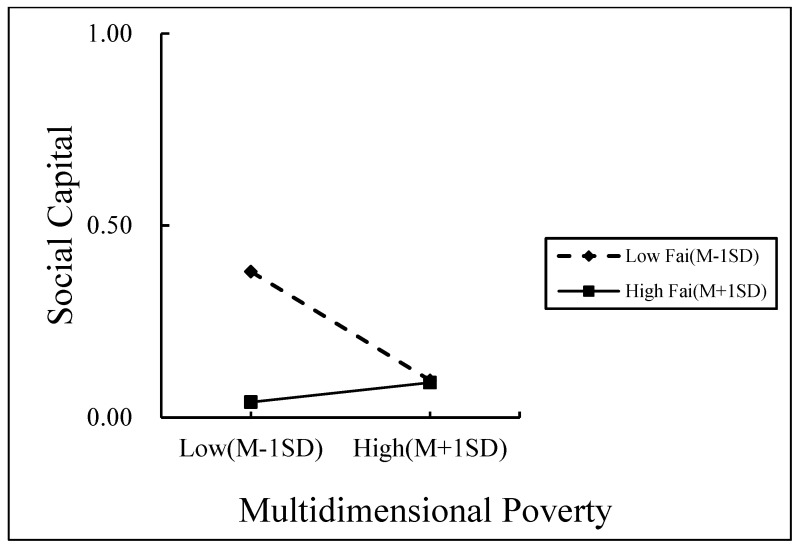
The moderating role of family care in the relationship between multidimensional poverty and social capital.

This was performed to show the moderating effect of family care more clearly (as shown in Figure 2 and Figure 3). According to Figure 2, social capital had a significant positive effect on rural households’ self-rated health for those with low levels of family care (simple slope = 1.742, t = 9.093, and *p* = 0.001), whereas for those with high levels of family care, multidimensional poverty had no significant impact (simple slope = 0484, t = 1.304, and *p* = 0.193). It demonstrated that the favorable impact of social capital on the rural household health increases with decreasing levels of family care. According to Figure 3, multidimensional poverty had a non-significant positive effect on rural households’ social capital levels when they provide high levels of family care (simple slope = 0.050, t = 1.554, and *p* = 0.121) but a significant negative effect when they provide low levels of family care (simple slope = −0.284, t = −4.721, and *p* = 0.001). It demonstrated that the detrimental impact of multidimensional poverty on the social capital of rural households increases with decreasing levels of family care.

## 5. Discussion and Conclusions

In the past, academic research and hot research topics have concentrated on alleviating and reducing income poverty as one of the major barriers to economic development in rural China. Though few studies have concentrated on the effects of relative poverty and multidimensional poverty on rural households’ health in the “post-poverty era,” the majority of the literature has addressed poverty and the impact of it on rural households’ lives from the perspective of income. In order to quantify the impact of multidimensional poverty on health and examine its mechanism, we used a sample of 382 out-of-poverty households in Jiangxi Province in 2020, with social capital as a mediating variable and family care as a moderating variable.

The contributions of this study were primarily reflected in the following three dimensions. First, in contrast to the majority of earlier studies, we looked at the multidimensional poverty effect from a variety of angles when choosing the poverty indicators for rural households. The income indicator was added and utilized as a separate dimension in addition to the three dimensions of health, education, and living standard in the MPI. While multidimensional relative poverty captures the sustainability of rural households, absolute poverty, assessed by income, merely shows the bare necessities of survival for rural households. In the sample area, rural poor households have been raised out of poverty on the basis of income alone, but according to the data from this study, 35% of these households have been lifted out of poverty but are still at risk of falling back into it on a multidimensional basis. We also measured the total social capital index of rural households. The entropy value of four indicators—social networks, social trust, social participation, and social support—was calculated to increase the variable’s representativeness when it came to the selection of the social capital variable for rural households. We took into account children under the age of 16 and elderly above the age of 60 who need assistive technology to do daily tasks when determining the degree of family care for rural households.

The self-rated health of rural households was typically shown to be very negatively impacted by multidimensional poverty. Poor rural households may be more susceptible to exposure to harmful physical health factors due to a lack of living resources, health awareness, and constrained income levels. As a result, the self-rated health of the rural households may be impacted. The results support both hypothesis 1 and earlier research, which is consistent with previous findings [17]. Due to a mix of chronic economic and life stress, a lack of resources, and subjective assessments of relative poverty, people suffer from reduced health.

The article indicated that multidimensional poverty of rural households had a positive impact on the self-rated health of the rural households primarily through social capital, which was employed as a mediating variable. That is, the negative impact of multidimensional poverty on the level of health in rural households was positively tempered by social capital. Social capital increases resource endowment and social engagement, this reduces the impact of multidimensional poverty on the self-rated health of rural households.

However, this study’s results differ from others [54], which do not consider the burden of non-labor resources in rural households, that is the level of family care in rural households. High-intensity family care implies that caregivers expend more energy and bear more opportunity costs (such as time expenses and leisure costs), which has an impact on rural households’ capacity to build up social capital. In other words, the role of social capital as a mediating factor in the impact of multidimensional poverty on the self-rated health of rural households is moderated by family care as a moderating variable. This affected the level of social capital of the rural households. The research showed that family care decreased the self-rated health of rural households and primarily evaluated that multidimensional poverty’s detrimental impact on rural households’ social capital increased with lower levels of family care. The impact of multidimensional poverty on the self-rated health of rural households becomes increasingly important as the level of family care rises. Social capital is less likely to affect the health of rural households by lowering their level of multidimensional poverty and subsequently their level of health as the amount of family care grows.

Second, to address how multidimensional poverty affected the self-rated health of rural households, our econometric research was based on a survey data and a moderated mediation model. The survey data allowed us to more effectively evaluated the question of the objectivity of indicators and, as a result, came to more trustworthy findings because both multidimensional poverty and social capital were measured from various aspects. Our research had shown that the likelihood of social capital attenuating the negative effects of multidimensional poverty on rural households’ self-rated health decreased as the burden of family care increased. In Jiangxi province of China, policy priority should be to increase the social capital of rural households [87].

Third, multiple effects of the same issue may be seen in the self-rated health of rural households. It has been found that social capital partially mediates the effect of multidimensional poverty on the levels of self-rated health in rural households [54]. However, we tested the mediating role of social capital in the effect of multidimensional poverty on the self-rated health of the rural households by examining the post-entropy value of social capital and found that social capital played a fully mediating role. Looking at multiple aspects of the household’s life helps us assess the level of social capital of a rural household as well as establish whether the household is experiencing multidimensional poverty. Thus, social capital plays a fully mediating role in the effect of rural households’ multidimensional poverty on self-rated health.

The conclusive disparities can be explained in several different ways. First, multidimensional social capital has not been taken into account in prior research. Meanwhile, the majority of earlier studies portrayed social capital using a one-dimensional model (such as social trust, social support, and social networks). However, this social capital is not strictly social capital because it is one-dimensional. In this study, we estimate the total index of social capital after the entropy value using the four dimensions: social networks, social trust, social participation, and social support. This is partially due to the fact that there are several ways in which various social capital categories and definitions might influence an individual’s degree of health. Second, earlier research used survey data from different geological terrains (such as the plains, hills, and mountains), yet terrain conditions, social conditions, and income levels in different locations will impact rural households’ subjective assessments of their social capital position. In this study, we also considered rural households in different conditions geological terrains as sample selection in order to classify the different effects of the geological factors.

These findings shed new light on the relationship between multidimensional poverty and rural households’ self-rated health. The level of family care is used as a moderating variable and social capital is used as a mediating variable following entropy in the article. A moderated mediating model is then used to test the hypothesis. This supports earlier findings that a greater portion of the influence of rural households’ self-rated health is explained by the mediating effect of social capital [53]. By enhancing our knowledge of the impact of multidimensional poverty on the self-rated health of rural households, this study adds to the body of literature. Additionally, the research uses mediated and moderated methods to identify variables linked to rural households’ self-rated health.

Even though the relationship between multidimensional poverty and the self-rated health of rural households has been better understood as a result of this study, there still exist some deficiencies that need further research.

First, the Jiangxi province of China has been the sole subject of this study. Thus, it is important to be cautious when interpreting our results. There should be more effort put into examining differences in the self-rated health of rural households between regions with various socioeconomic and cultural characteristics. At the same time, a lot of indicators and data are needed for the scientific measurement of health levels, and there is still a significant gap between the indicators used by the academic community and the medical health measurement technology. More abundant and high-quality data will be needed to measure rural households’ health levels more accurately and scientifically.

Second, because social capital is a broad concept, there are other ways to categorize it, including a taxonomy of individual and group social capital, bonded and linked social capital, and more. Further research is necessary to fully understand the various impacts of various social capital on the health of rural populations. However, family care is simply one of the variables that affect rural households’ social capital and multidimensional poverty. Future research should look at how these and other aspects of poverty affect health in order to fully understand the effects of multidimensional poverty on the self-rated health of rural households. 

Third, as a limitation of expanding the research, it is also necessary to account for cultural aspects as social care may not be typical in some countries. Additionally, the social care is a wide concept, which has many other dimensions, such as the social care structure and age makeup. In order to fully capture the impacts of social care on the rural household’s health, it is worthwhile for future research to further explore the impact of the elements of cultural aspects on the rural household’s behavior.

This study aimed to address how the multidimensional poverty affected the self-rated health of rural households. Social capital was used as a mediating variable and family care was used as a moderating variable. We conducted the model and used the survey data including 382 out-of-poverty sample rural households from 2020 in Jiangxi Province, enabling us to take advantage of the moderated mediation model in exploring the relationship between multidimensional poverty and the self-rated health. It was revealed that multidimensional poverty affected rural households’ self-rated health primarily through social capital (β = 3.240 and t = 13.482) and that family care moderated the mediating pathway. Family care exacerbated the negative effect of multidimensional poverty on rural households’ self-rated health (β = 0.558 and t = −5.221) and weakened the beneficial effect of social capital on rural households’ self-rated health (β = −2.100, t = −3.304). The lower the level of family care, the more significant the positive effect of social capital on rural households’ health (simple slope = 1.742, t = 9.093, and *p* = 0.001).

To strengthen the viability of rural households in response to the fundamental needs of rural areas in the research region, targeted policies for reducing poverty should be developed. China should rely on the current information system for poverty alleviation and development, consider elements such as medical care, education, income, and living conditions, summarize the experience and failures of the period of eradicating poverty, and establish reasonable income baseline and vulnerability line monitoring standards in light of the current situation. Firstly, concentrate on boosting infrastructure building and medical and healthcare investment in rural areas. To stop and slow the spread of diseases, improve the medical and health infrastructure in rural areas. Secondly, in addition to strengthening the fight against adult illiteracy, literature that is tailored to farmers’ requirements should be published, and cultural events should be planned to raise the literacy levels of farm households. The development of social capital in rural households, leveraging social capital’s contribution to eradicating multifaceted poverty, and assisting farmers in deepening and broadening their interpersonal connections should then be the focus of attention. Finally, in order to lighten the pressure on family care and lessen the burden on family carers, the long-term care insurance system should be reinforced to encourage and support social care as well as the development of care offered by social institutions and community services. The government must consistently encourage the growth of socialized care services to fill the gap in family care so that social care can be used as a complementary or alternative resource to family care.

## Figures and Tables

**Table 1:** Dimensions, indicators, cutoffs, and weights of the MPI.

Dimension	Indicator (Relative Weight)	Deprived If...	Cutoffs
Health	Medical expenses (1/8)	Medical expenses incurred by a member of the family suffering from a serious illness or hospitalization	Qualitative indicator: 1 = poor; 0 = non-poor
	Health insurance (1/8)	A member of the family does not have rural health insurance	Qualitative indicator: 1 = poor; 0 = non-poor
Education	Years of education (1/8)	A member of the family who are 16 years of age or older and have less than 9 years of education.	9
	Child enrollment (1/8)	School enrollment expenditures greater than 40% of the national median per capita disposable income of rural residents in 2019	5755.6
Income	Per capita disposable income (1/4)	Household disposable income per capita is less than 40% of the national median disposable income per capita for rural residents in 2019	5755.6
Living Standard	Electricity (1/20)	No electricity in the home	Qualitative indicator: 1 = poor; 0 = non-poor
	Cooking fuel (1/20)	The primary fuel for cooking is unclean fuel	Qualitative indicator: 1 = poor; 0 = non-poor
	Floor (1/20)	The structure is made of mud	Qualitative indicator: 1 = poor; 0 = non-poor
	Assets (1/20)	Households that do not own more than one of the following assets: battery car, car, television, Refrigerator, washing machine, telephone, air conditioner, computer, electric heater, or water heater	Qualitative indicator: 1 = poor; 0 = non-poor
	Per capita housing area (1/20)	Less than 12 square meters are seen as a sign of deprivation in terms of housing area per capital.	12

**Table 2:** Variable description.

Variables	Definition	Min	Max	Mean	SD
Dependent variable					
Health	Self-rated health level; 1 = very poor, 2 = poor, 3 = average, 4 = good, 5 = very good	1	5	2.510	1.317
Independent variable					
Multidimensional poverty	Multidimensional poverty rural households; 1 = multidimensional poverty, 0 = non-multidimensional poverty; binary variables	0	1	0.350	0.477
Mediating variable					
Total social capital index	Social capital after entropy value	0	0.903	0.225	0.235
Social networks	How many relatives in your family are village, township cadres or other public officials; continuous variable	0	2	0.235	0.553
Social trust	Do you believe in the government; 1 = very trusting, 2 = trusting, 3 = general, 4 = distrustful, 5 = very distrustful	1	5	3.298	1.199
Social participation	Whether your family participates in organizations such as planting associations and cooperatives; 1 = yes,0 = no; binary variables	0	1	0.397	0.490
Social support	How many people will come to help if the family holds a red and white wedding; continuous variable	0	90	14.505	14.008
Moderating variable					
Family care	Proportion of the total number of children under 16 years of age and elderly people over 60 years of age in the household who need assistance with their daily lives to the total household size; continuous variable	0	1	0.458	0.299
Individual characteristics					
Gender	Gender of rural household; 1 = male, 0 = female; binary variables	0	1	0.821	0.383
Age	Age of rural household; continuous variable	18	92	59.185	14.941
Education	Years of education of rural household; continuous variable	0	16	4.229	3.386
Marriage	Marital Status; 1 = unmarried, 2 = first marriage, 3 = remarried, 4 = divorced, 5 = widowed	1	5	2.583	1.438
Household characteristics					
Number of family	Continuous variable	1	7	2.850	1.577
Number of labor force	Continuous variable	0	5	0.926	1.057
Income	Logarithm	0	11.51	8.831	3.352
Number of samples	382				

**Table 3:** Mean comparison between multidimensional poverty and non-multidimensional poverty group.

Variables	Multidimensional Poverty Group (*n* = 134)		Non-Multidimensional Poverty Group (*n* = 248)		T-Value
	Mean	SD	Mean	SD	
Dependent variable					
Health	1.990	0.914	2.790	1.416	6.669 ***
Mediating variable					
Total social capital index	0.099	0.118	0.293	0.254	10.155 ***
Social networks	0.040	0.208	0.340	0.647	6.553 ***
Social trust	2.890	1.122	3.520	1.183	5.073 ***
Social participation	0.150	0.358	0.530	0.500	8.645 **
Social support	14.720	13.945	14.390	14.069	−0.214
Moderating variable					
Family care	0.736	0.170	0.308	0.241	−20.120 ***
Individual characteristics					
Gender	0.860	0.350	0.800	0.399	−1.360
Age	58.340	15.527	59.641	14.627	0.810
Education	3.570	3.004	4.580	3.531	2.817 **
Marriage	2.600	1.388	2.580	1.468	−0.132
Household characteristics					
Number of family	3.130	1.612	2.700	1.541	−2.601 *
Number of labor force	1.100	1.178	0.830	0.975	−2.431 *
Income	6.491	4.609	10.105	1.150	8.901 ***

Note: * *p* < 0.1, ** *p* < 0.05, *** *p* < 0.01.

**Table 4:** Regression analysis of the mediating role of social capital.

	R	R2	F (df)	β	t
***Model 1***					
Outcome: Health					
Predictors: Gender	0.392	0.153	8.468 (8)	−0.265	−1.405
Age				0.016	3.433 ***
Education				0.051	2.510 **
Marriage				0.088	1.716 *
Number of family				−0.055	−1.023
Number of labor force				0.141	1.755 *
Income				−0.007	−0.328
Multidimensional Poverty				−0.751	−4.775 ***
***Model 2***					
Outcome: Social Capital					
Predictors: Gender	0.413	0.171	9.639 (8)	−0.035	−1.066
Age				0.000	0.102
Education				0.002	0.806
Marriage				0.013	1.485
Number of family				0.002	0.229
Number of labor force				0.002	0.177
Income				−0.001	−0.433
Multidimensional Poverty				−0.197	−7.08 ***
***Model 3***					
Outcome: Health					
Predictors: Gender	0.656	0.431	31.372 (9)	−0.150	−0.967
Age				0.016	4.112 ***
Education				0.042	2.493 **
Marriage				0.044	1.051
Number of family				−0.062	−1.407
Number of labor force				0.132	2.015 **
Income				−0.008	−0.097
Multidimensional Poverty				−0.113	−0.822
Social Capital				3.240	13.482 ***

Note. Bootstrap sample size = 5000. LL = low limit, CI = confidence interval, UL = upper limit. * *p* < 0.1. ** *p* < 0.05. *** *p* < 0.01.

**Table 5:** Decomposition of total effect, direct effect, and mediating effect.

	Effect	BootSE	BootLLCI	BootULCI	Effect Ratio
Mediating effect	−0.638	0.083	−0.807	−0.480	84.95%
Direct effect	−0.113	0.132	−0.370	0.151	15.05%
Total effect	−0.751	0.137	−1.026	−0.484	

**Table 6:** Mediating effects test with moderation.

	R	R2	F (df)	β	t
***Model 4***					
Outcome: Social Capital					
Predictors: Multidimensional Poverty	0.616	0.380	22.699 (10)	−0.373	−4.949 ***
Family Care				−0.568	−11.271 ***
Multidimensional Poverty*Family Care				0.558	−5.221 ***
Gender				−0.015	−0.520
Age				−0.001	−1.503
Education				−0.001	−0.445
Marriage				0.007	0.957
Number of family				0.006	0.832
Number of labor force				−0.001	−0.102
Income				−0.002	−0.667
***Model 5***					
Outcome: Health					
Predictors: Multidimensional Poverty	0.861	0.742	96.824 (11)	1.0594	9.482 ***
Family Care				−3.428	−15.17 ***
Social Capital				2.079	8.575 ***
Social Capital*Family Care				−2.100	−3.304 ***
Gender				−0.076	−0.721
Age				0.004	1.782
Education				0.015	1.375
Marriage				0.010	0.367
Number of family				−0.015	−0.499
Number of labor force				0.085	1.926 *
Income				−0.014	−1.180

Note. Bootstrap sample size = 5000. LL = low limit, CI = confidence interval, UL = upper limit. * *p* < 0.1. *** *p* < 0.01.

**Table 7:** Direct and mediating effects at different levels of family care.

	Family Care	Effect	BootSE	BootLLCI	BootULCI
Mediating effects with moderation	eff1 (M − 1SD)	−0.494	0.085	−0.670	−0.334
	eff2 (M)	−0.130	0.042	−0.219	−0.057
	eff3 (M + 1SD)	0.025	0.025	−0.016	0.084
Comparison of mediating effects with moderation	eff2-eff1	0.364	0.060	0.250	0.488
	eff3-eff1	0.519	0.091	0.346	0.709
	eff3-eff2	0.154	0.054	0.049	0.259

## Data Availability

Not applicable.
